# Protective Effect of Irisin on Atherosclerosis via Suppressing Oxidized Low Density Lipoprotein Induced Vascular Inflammation and Endothelial Dysfunction

**DOI:** 10.1371/journal.pone.0158038

**Published:** 2016-06-29

**Authors:** Yuzhu Zhang, Qian Mu, Zheng Zhou, Haibo Song, Yuan Zhang, Fei Wu, Miao Jiang, Fang Wang, Wen Zhang, Liang Li, Lei Shao, Xingli Wang, Shiwu Li, Lijun Yang, Qi Wu, Mingxiang Zhang, Dongqi Tang

**Affiliations:** 1 Center for Stem Cell and Regenerative Medicine, The Second Hospital of Shandong University, Jinan, People’s Republic of China; 2 Chaohu Road Community Health Center of Qingdao, Qingdao, People’s Republic of China; 3 Key Laboratory of Cardiovascular Remodeling and Function Research, Qilu Hospital of Shandong University, Jinan, People’s Republic of China; 4 Department of Pathology, Immunology, and Laboratory Medicine, University of Florida College of Medicine, Gainesville, Florida, United States of America; 5 Department of Anatomy, School of Medicine Shandong University, Jinan, People’s Republic of China; University of Hull, UNITED KINGDOM

## Abstract

Irisin, a newly discovered myokine, is considered as a promising candidate for the treatment of metabolic disturbances and cardiovascular diseases. In the present study, we used two animal models, apolipoprotein E-deficient mice fed on a high-cholesterol diet and a mouse carotid partial ligation model to test the anti-atherosclerotic effect of irisin. Irisin treatment (0.5 μg/g body weight/day) significantly reduced the severity of aortic atherosclerosis in apolipoprotein E-deficient mice fed on a high-cholesterol diet and suppressed carotid neointima formation in a carotid partial ligation model. It was associated with decreased inflammation and cell apoptosis in aortic tissues. In addition, in a cell culture model, irisin restored ox-LDL-induced human umbilical vein endothelial cell dysfunction by reducing the levels of inflammatory genes via inhibiting the reactive oxygen species (ROS)/ p38 MAPK/ NF-κB signaling pathway activation and inhibiting cell apoptosis via up-regulating Bcl-2 and down-regulating Bax and caspase-3 expression. Our study demonstrated that irisin significantly reduced atherosclerosis in apolipoprotein E-deficient mice via suppressing ox-LDL-induced cell inflammation and apoptosis, which might have a direct therapeutic effect on atherosclerotic diseases.

## Introduction

Atherosclerosis, the primary cause of coronary artery disease and stroke, is the leading cause of death in the developed world [1, 2]. The pathophysiological mechanisms and the etiology of atherosclerosis are rather complex [3], among which endothelial dysfunction and inflammation play a critical role [4]. Various risk factors can induce endothelial injury which contributes to the initiation and progression of atherosclerosis [1, 5–7]. The inflammatory responses to endothelial damage are followed by the development of vascular lesions in the blood vessels [8]. Therefore, prevention of endothelial injury could be an important therapeutic target in the treatment of atherosclerotic cardiovascular diseases.

Skeletal muscle has been identified as an endocrine organ in recent years [9]. Cytokines secreting by skeletal muscle during or immediately after exercise [10, 11], known as myokines, mediate some of the beneficial effect of exercise on metabolism and the cardiovascular system. Irisin, a newly discovered myokine containing 111 amino acids with a molecular mass of 22 kDa, is released upon cleavage of the membrane protein fibronectin type III domain-containing protein 5 (FNDC5). Muscle FNDC5 gene expression is induced by peroxisome proliferator-activated receptor-γ co-activator-1α, which is a transcriptional co-activator in the muscle that is up-regulated following 3 weeks of free wheel running in mice [12, 13]. A recent study demonstrated the presence of human irisin in blood using quantitative mass spectrometry [14]. Additionally, recent studies discovered that type-2 diabetes mellitus, insulin resistance, and the metabolic syndrome are associated with reduced irisin levels [15, 16]. Meanwhile, some controversial results have recently been reported, such as obesity having a positive correlation with circulating irisin levels [17]. The controversial results raise serious concerns about its prospective application. Further studies are needed to resolve the concerns that have been raised about the application of irisin. Circulating irisin levels are positively associated with endothelium-dependent arterial dilation [18]. Also, our recent study showed that irisin promotes human umbilical vein endothelial cell (HUVEC) survival and proliferation via the ERK signaling pathway [19]. However, the effects of irisin on the formation of atherosclerosis and its underlying mechanisms are not fully understood.

In the present study, we reported that irisin prevented the formation of atherosclerotic lesion in apolipoprotein E (Apo E)-deficient mice and improved vascular remodeling in the mouse carotid ligation model. Using *in vitro* experiments, we demonstrated that irisin could attenuated ox-LDL induced endothelial cell inflammation via inhibition of the ROS/ p38 MAPK/ NF-κB signaling pathway, and alleviated ox-LDL induced endothelial cell apoptosis by up-regulating Bcl-2 expression and down-regulating Bax and caspase-3 expression.

## Methods

### Expression and purification of human irisin

The expression and purification of irisin was performed as previously described [13]. Briefly, human irisin cDNA (360bp) was designed, synthesized (Life Technologies, USA), and then cloned into the EcoR1/Xba1 sites of the pPICZαA plasmid. After linearizing, the pPICZaA-irisin plasmid was transformed into Pichia pastoris X-33 according to the kit manual (Pichia Easycomp Transformation Kit; Invitrogen). The culture of yeast and induction of protein expression were performed as previously described [13]. The recombinant irisin (r-irisin) protein in the supernatant was purified by a two-step method [13]. First, 60% saturated ammonium sulfate was used to precipitate the r-irisin. The precipitated proteins were collected, dissolved in buffer A (25 mM Hepes pH 7.9, 10% glycerol, 0.1 M KCl, 0.2 mM EDTA, and 0.5 mM DTT), and then dialyzed against the same buffer. The resulting sample was loaded onto a ConA-agarose column. The column was first washed with 0.2 M KCl in buffer A, and proteins were eluted with 1.5M KCl in buffer A. The elution fractions were analyzed by western blotting. The purified irisin was dialyzed against 10% glycerol in normal saline (NS) and stored at -80°C. To determine the purity of irisin, the secreted r-irisin was separated by SDS-PAGE and stained with coomassie brilliant blue. There were three protein bands present on the gel with molecular weight of 25 kD, 22 kD and 15 kD (S1 Fig).

### Mouse models

Male Apo E-deficient mice (6–8 weeks old) purchased from Vital River Laboratory Animal Technology Co., Ltd (China) were fed with a high cholesterol diet (HCD) containing 21% fat and 0.15% cholesterol. Animals were randomly divided into two groups, control (*n* = 8) and irisin-treatment (*n* = 8). In the irisin treatment group, the mice were treated daily with purified irisin at a dose of 0.5 μg/g body weight (a dose determined from our previous report) [13] by intraperitoneal (i.p.) injection for the last 8 weeks of a 12-week HCD feeding program. In the control group, the mice were given normal saline in the same manner. At the end of irisin treatment, mice were anesthetized with intraperitoneal injections of 50 mg/kg pentobarbital sodium (Nembutal sodium solution, Wuhan Entai Technology, Wuhan, China), and then aortas, aortic roots, and carotid arteries were taken from the mice.

For the neointima study, male Apo E-deficient mice (6–8 weeks old) were anesthetized with intraperitoneal injections of 50 mg/kg pentobarbital sodium. Partial ligation of the left common carotid artery was carried out as previously described [20]. Briefly, a ventral midline incision (4–5 mm) was made in the neck, and the left carotid artery was exposed by blunt dissection. The left external and internal carotid and occipital arteries were ligated with a 6–0 silk suture while the superior thyroid artery was left intact to allow blood outflow. 24 hours after partial ligation, the mice were randomly divided into two groups, control (*n* = 5) and irisin-treatment (*n* = 5). In the irisin treatment group, the mice were treated daily with purified irisin at a dose of 0.5 μg/g body weight by i.p. injection for 4 weeks. In the control group, the mice were given NS in the same manner. All of the animals were fed with a HCD. At the end of irisin treatment, mice were anesthetized with intraperitoneal injections of pentobarbital sodium, blood samples were collected and then the segments of the left carotid artery just proximal to the ligation were excised.

### Ethics statement

Animals were housed individually in cages in a temperature and humidity-controlled, pathogen-free environment, with a 12 h light/dark cycle. All animals received food and water *ad libitum*. The physiological condition of animals was monitored daily and each mouse was weighed weekly. Sustained weight loss, disinterest in eating or drinking, abnormal respirations, significant dermatitis not responding to adequate treatment, significant lethargy, and moribund appearance were defined as humane endpoints. These animals were excluded from the experiment and euthanized. In total, 12 mice underwent carotid artery partial ligation but 2 mice died of unknown causes the day after successfully recovering from surgery. They did not show any clinical signs of ill health and even autopsy did not reveal the cause of death, therefore they were not considered for further analyses. No other animals met the criteria for euthanasia prior to reaching designated time points. When euthanized, the mice were anesthetized with the intraperitoneal injection of pentobarbital sodium and then sacrificed by cervical dislocation. All animal experiments were carried out in accordance with the National Institutes of Health Guide for the Care and Use of Laboratory Animals and were approved by the Shandong University Laboratory Animals Care and Use Committee. All animal experiments were carried out in accordance with the National Institutes of Health Guide for the Care and Use of Laboratory Animals and were approved by the Shandong University Laboratory Animals Care and Use Committee.

### Evaluation of aortic atherosclerotic lesions

For en face quantification of atherosclerotic lesions, the aorta was dissected from 1 mm above the aortic valve to the iliac bifurcation. For atherosclerotic lesion examination, the aorta was stained with Oil-Red O (Sigma). The images of aortas were captured with a video camera connected to a Leica MZ 10F dissection microscope, and analyzed using Image Pro Plus software (Version 6.0; Media Cybernetics).

For quantification of atherosclerotic lesions in the aortic root, serial frozen sections (10 μm thickness) of the aortic root were collected. Sections were first stained with Oil-Red O and then counterstained with hematoxylin. Images were captured using a Nikon Eclipse Ti microscope, and then the atherosclerotic plaque area was quantified by use of Image Pro Plus software (Version 6.0; Media Cybernetics). The mean lesion area was quantified in 5 equally spaced aortic root sections per mouse.

### Morphometry

The carotid arteries were harvested and embedded in paraffin. Five cross sections (5 μm thickness) per animal were cut at 100 μm intervals, stained with hematoxylin and eosin. Images were captured using a Nikon eclipse Ti microscope, and then analyzed using Image Pro Plus software (Version 6.0; Media Cybernetics). The neointima area was designated as the area defined by the luminal surface and internal elastic lamina. The medial area was defined by the internal elastic lamina and external elastic lamina. The N/M ratio was expressed as the ratio of the neointima area to media area.

### Measurement of lipid parameters in the serum

The concentrations of total cholesterol (TC), triacylglycerol (TG), low-density lipoprotein cholesterol (LDL-C), and high density lipoprotein cholesterol (HDL-C) in the serum were measured by enzymatic colorimetric assays using commercially available detection kits (Biosino Biotechnology Co., Ltd, Beijing, China).

### Immunohistochemical staining

Immunohistochemical staining was performed using GTVisionTM ⅢDetection System/Mo&Rb (GK500705,Gene Tech). Paraffin-embedded sections were stained with primary antibodies for CD3 (1:100, ab16669, Abcam) and CD68 (1:200, ab955, Sigma). After washing, sections were incubated with horseradish peroxidase-conjugated secondary antibodies. Immunocomplexes were then detected using diaminobenzidine tetrahydrochloride dihydrate substrate. Images were captured using a Nikon Eclipse Ti fluorescent microscope, and the stained area was measured using Image Pro Plus software (Version 6.0; Media Cybernetics).

### Enzyme-linked immunosorbent assay (ELISA)

The serum concentrations of ICAM-1, VCAM-1, IL-6, and MCP-1 in mice were measured by ELISA assays (Boster, Wuhan, China) according to the manufacturer’s instructions. The color absorbance at 450 nm was measured using a Bio-Rad microplate reader.

### TUNEL staining

Apoptotic cells in atherosclerotic plaque were detected by terminal deoxynucleotidyl transferase (TdT)-mediated dUTP nick end labeling (TUNEL) using a kit (R&D Systems) according to the manufacturer’s instructions. After TUNEL labeling, sections were counterstained with 4’-6-diamidino-2-phenylindole to detect nuclei.

### Cell culture

HUVECs were isolated from human umbilical cords using 300 units/ml collagenase II (Sigma-Aldrich, St. Louis, MO). Cells were cultured in Medium 199 (Invitrogen) supplemented with 10% (v/v) fetal bovine serum (FBS, Invitrogen), 10 ng/mL EGF (Peprotech), and 10 ng/mL bFGF (Peprotech) at 37°C in a humidified atmosphere containing 5% CO_2_. HUVECs were used between passages 3 and 7. All experiments were carried out with the same batch of HUVECs, which were from a single donor. The murine RAW 264.7 macrophage cells (ATCC, TIB-71) were cultured in DMEM supplemented with 10% (v/v) fetal bovine serum (FBS, Invitrogen) at 37°C in a humidified atmosphere containing 5% CO_2_. The study protocol conformed to the ethical guidelines of the 1975 Declaration of Helsinki with the approval of the Institutional Medical Ethics Committee of Qilu Hospital, Shandong University. All of the donors provided written informed consent.

### Cell viability assay

The methyl thiazolyl tetrazolium (MTT; Sigma, USA) assay was used to evaluate the cell viability according to the manufacturer’s instructions. Briefly, HUVECs (1 ×10^4^ cells/well) were plated in a 96-well plate and treated with different concentrations of ox-LDL (Yiyuan Biotechnologies, Guangzhou, China) or irisin for 24 h. Then, 20 μL MTT (5mg/mL) was added to each well for 4 h incubation. After incubation, dimethylsulfoxide was added to each well. The absorbance at 490 nm was determined using a microplate reader (Bio-Rad, Hercules, CA). For each group, 6 duplicate wells were detected per experiment.

### Quantitative RT-PCR

Total RNA from homogenized tissues or cells was extracted using TRIzol® reagent (Invitrogen) following the standard protocol, and reverse transcription reactions (SuperScript III First-Strand Synthesis System for RT-PCR, Invitrogen Corp., Carlsbad, CA) were performed with 2 μg of total RNA. Quantitative real time PCR (qPCR) was carried out in triplicate with the SYBR Green Master Mix (Applied Biosystems) using the Bio-Rad CFX96 Real-Time System (Bio-Rad, Hercules, CA). The *2*^*-ΔΔct*^ method was used to calculate the relative expression of genes using beta-actin RNA as an internal control. Primers used for qPCR are provided in S1 Table.

### Intracellular reactive oxygen species (ROS) measurement

Intracellular ROS generation was evaluated by 2’, 7’-dichlorofluorescein diacetate (DCFH-DA) as previously described [21]. Briefly, after exposure to ox-LDL and/or irisin for 24 h, HUVECs were treated with 10 μM DCFH-DA for 20 min at 37°C. After washing the cells three times with PBS, the fluorescence intensity was detected with a multi-detection microplate reader with excitation at 488 nm and emission at 530 nm within 15 min. The measured fluorescence values were expressed as a percentage of the fluorescence in control cells.

### Western blot

Total cellular protein was isolated using RIPA buffer (150 mM NaCl, 50 mM Tris (pH 8.0), 150 mM NaCl, 1% NP-40, 0.5% sodium deoxycholate, and 0.1% SDS) containing 1 mM Na_3_VO_4_, 5mM NaF and protease inhibitor cocktail (Roche). The nuclear protein and cytoplasmic proteins of macrophages were extracted with a nuclear and cytoplasmic protein extraction kit (Beyotime, China). The protein concentration was determined using the BCA protein assay kit (Pierce, Rockford, IL, USA). Proteins were detected using the following primary antibodies: phospho- NF-κB p65 (1:1000, sc-33020, Santa Cruz), NF-κB p65 (1:1000, sc-372, Santa Cruz), phospho-p38 MAPK (1:1000, 9211, Cell Signaling), p38 MAPK (1:1000, 9212, Cell Signaling), caspase-3 (1:1000, 9662, Cell Signaling), Bax (1:1000, 5023, Cell Signaling), Bcl-2 (1:1000, 2870, Cell Signaling), Histon H3 (1:2000, ab52946, Abcam), and glyceraldehyde 3-phosphate dehydrogenase (GAPDH, 1:1000, sc-25778, Santa Cruz). After incubation with HRP-conjugated secondary antibody (1:5000, Santa Cruz) at room temperature for 1 hour, the immune complexes were detected using the ECL method. Densitometric analysis was performed using Alpha Imager 2200.

### Apoptosis analysis

HUVECs were seeded into 6-well plates. When cells reached 80% confluence, they were incubated with or without 20 nM irisin and 80 μg/mL ox-LDL for 24 hours. Apoptotic cells were determined by using an Annexin V-FITC apoptosis detection kit (BD Biosciences, USA) according to the manufacturer’s instructions. Briefly, the cells were washed twice with cold PBS and then re-suspended. Subsequently, cells were incubated for 15 minutes at room temperature in the dark in 100μl binding buffer containing 5 μl Annexin V-FITC and 5 μl PI. Cellular fluorescence was analyzed by the BD accuriC6 flow cytometer using FlowJo (FlowJo, Ashland, OR, USA) software.

### Foam cell formation

When RAW 264.7 cells (ATCC, TIB-71) reached 80% confluence, they were incubated with or without 20 nM irisin and 80 μg/mL ox-LDL (Yiyuan Biotechnologies, Guangzhou, China) for 24 hours. Cells were then fixed with 4% w/v paraformaldehyde for 30 min and stained with 0.3% Oil-Red O for 15 min. Images were captured using a Nikon Eclipse Ti fluorescent microscope, and the Image Pro Plus software was used for the quantitative analysis of the lipid accumulation in the foam cells.

### Statistical analysis

All experiments were performed at least three times. Data are expressed as the mean ± SEM. Statistical comparisons were made between two groups with the *t*-test and between multiple groups by ANOVA. *P* values < 0.05 were considered to be statistically significant.

## Results

### Irisin inhibits atherosclerotic development and neointima formation in Apo E-deficient mice

**Fig 1:**
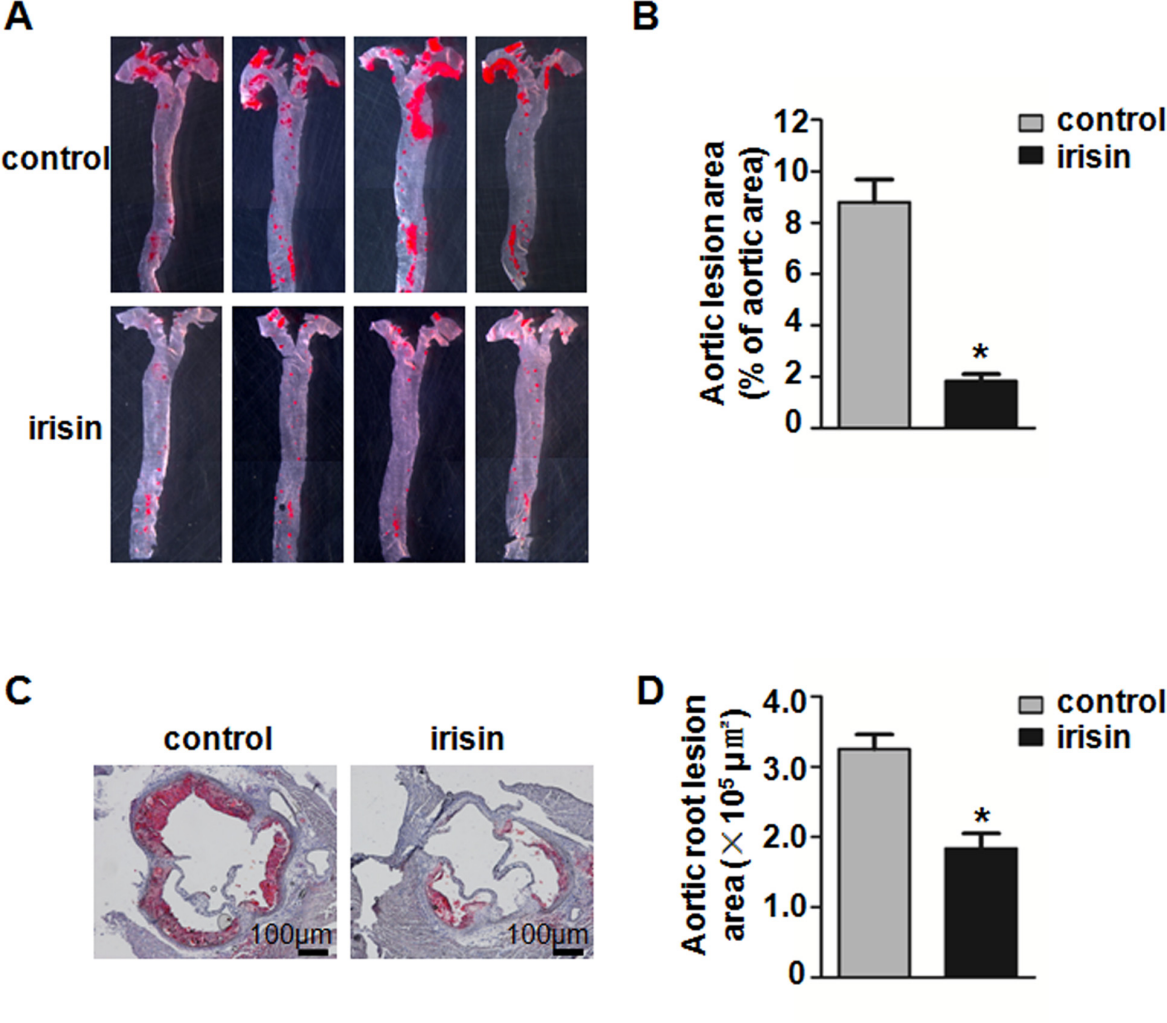
Influence of irisin on atherosclerotic lesion formation in Apo E-deficient mice. Apo E-deficient mice were treated with irisin for the last 8 weeks of a 12-week HCD feeding program (*n* = 8). (A) The atherosclerotic lesions in en face-prepared aorta were identified with Oil-Red O staining. (B) Lesion area (%) was expressed as percentage of atherosclerotic area/total area of the aorta. (C) The lipid-rich atherosclerotic lesions in aortic sinus were identified with Oil-Red O staining. (D) Quantification of atherosclerotic lesion areas in aortic root sections. *P < 0.05 vs. control, the data was expressed as the mean ± SEM.

We used two animal models, ApoE-deficient mice and a carotid partial ligation model, to study the effect of irisin in atherosclerosis and neointima formation. In ApoE-deficient mice, irisin treatment for 8 weeks significantly reduced atherosclerotic lesions on the surface of en face prepared aorta (1.84 ± 0.35% in irisin-treated mice *vs*. 8.80 ± 0.87% in NS-treated mice) (Fig 1A and 1B) and cross sections of the aortic root (1.84 ± 0.2×10^5^ μm^2^ in irisin-treated mice vs. 3.25 ± 0.20×10^5^ μm^2^ in NS-treated mice) (Fig 1C and 1D) stained with Oil-Red O. However, there were no significant differences in blood pressure, serum TC, TG, LDL-C, and HDL-C levels between the NS-treated and irisin-treated groups (S2 Table).

**Fig 2:**
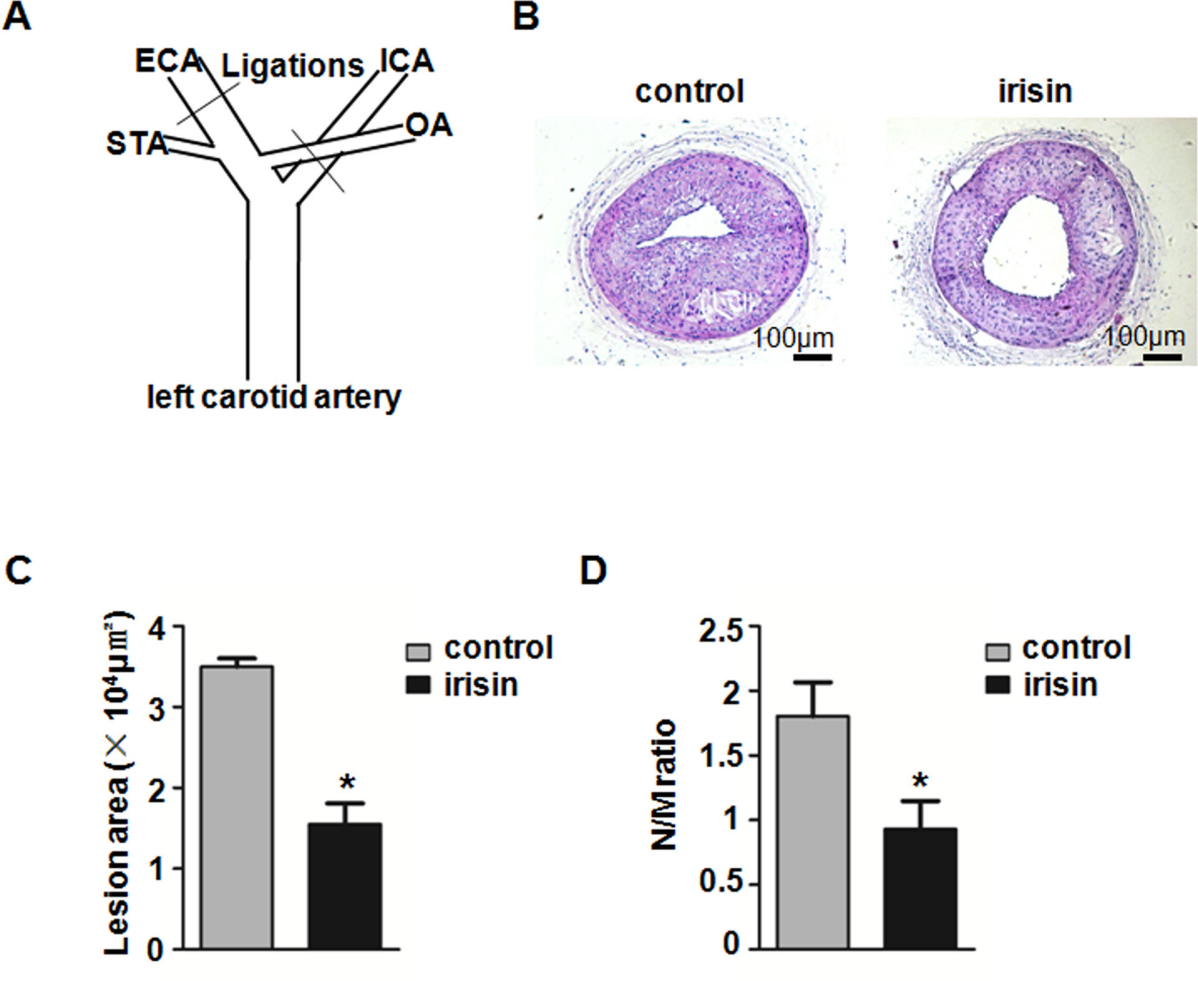
Influence of irisin on neointima formation in partial ligated carotid arteries. Apo E-deficient mice were treated with or without irisin for 28 days after carotid artery partial ligation (*n* = 5). (A) Scheme of partial ligation of the left carotid artery is shown. The left external carotid artery (ECA), internal carotid artery (ICA), and occipital artery (OA) were ligated with 6–0 silk suture. After ligation, flow was maintained only in the superior thyroid artery (STA). (B) Cross-sections of carotid arteries were identified with hematoxylin–eosin staining. (C) Quantification of the neointima areas of the partially ligated carotid arteries is shown. (D) Degree of neointima formation 28 days after carotid ligation was calculated according to N/M ratio. *P < 0.05 vs. control. The data was expressed as the mean ± SEM.

In the carotid partial ligation model (Fig 2A), the neointima formation in Apo E-deficient mice treated with irisin (1.55 ± 0.26×10^4^ μm^2^) for 4 weeks was significantly decreased compared with control subjects (3.50 ± 0.10×10^4^ μm^2^) receiving NS (Fig 2B and 2C). The ratio of the neointima area to media area (N/M ratio) was also decreased in the irisin treatment group (0.93 ± 0.04) 4 weeks after surgery compared with the control group (1.80 ± 0.26) (Fig 2D). These results suggest that irisin can inhibit atherosclerosis and neointima formation in Apo E-deficient mice.

### Irisin suppresses inflammation in partial ligated carotid arteries

**Fig 3:**
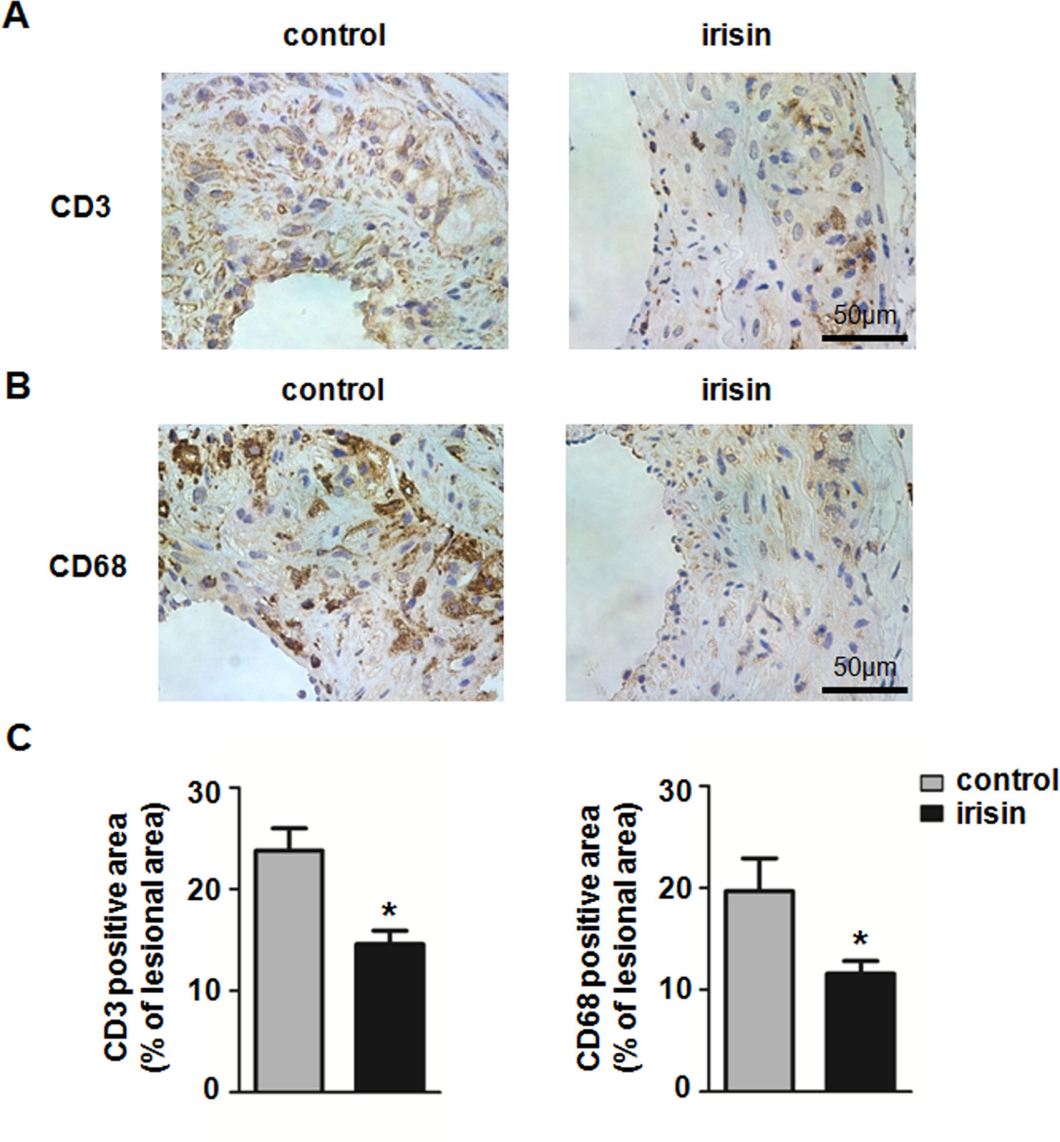
Influence of irisin on the inflammatory infiltration in partial ligated carotid arteries. Representative pictures of immunohistochemistry staining of CD3 (A), and CD68 (B) in partial ligated carotid arteries are shown. Brown staining exhibits positive area, while blue represents counterstaining with hematoxylin. (C) Quantification of positive staining areas in carotid artery sections is shown. *P < 0.05 vs. control, *n* = 5 per group. The data was expressed as the mean ± SEM.

**Fig 4:**
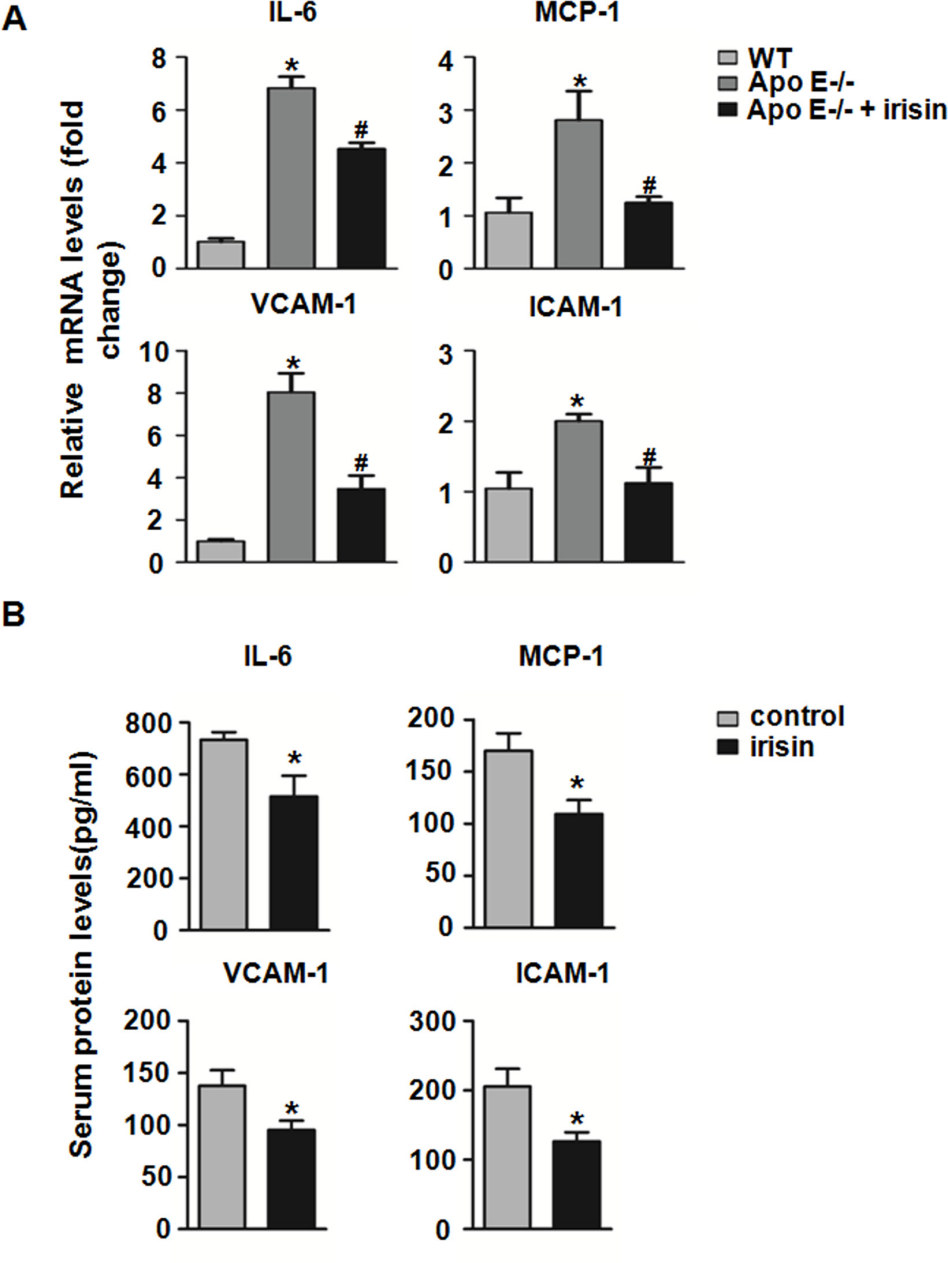
Influence of irisin on the inflammatory genes in partial ligated carotid arteries. (A) The mRNA expressions of IL-6, MCP-1, ICAM-1, and VCAM-1 in carotid arteries after partial ligation were analyzed by qPCR. The mRNA levels were normalized to that of β-actin. (B) The concentrations of IL-6, MCP-1, ICAM-1, and VCAM-1 protein in plasma were measured by ELISA. *P < 0.05 *vs*. WT mice, ^**#**^ *P <* 0.05 *vs*. Apo E-deficient mice treated with NS, *n* = 5 per group. The data was expressed as the mean ± SEM.

Since inflammation is postulated to play a critical role in the atherogenesis, we further assessed the inflammatory status in mice treated with irisin injections. We analyzed the effects of irisin on the inflammatory infiltration in atherosclerotic lesions by immunohistochemistry. The areas that were CD3 and CD68 positive were significantly reduced after irisin treatment (Fig 3A and 3B). Next, the expression of inflammatory genes in injured arteries was assessed. We found that interleukin-6 (IL-6), macrophage chemoattractant protein-1 (MCP-1), intercellular cell adhesion molecule-1 (ICAM-1), and vascular cell adhesion protein 1 (VCAM-1) mRNA expression in plaque lesions of the partial ligated carotid artery were significantly decreased compared to the control group treated with a saline injection (Fig 4A). In addition, ELISA showed that the protein levels of serum ICAM-1, VCAM-1, IL-6, and MCP-1 were significantly decreased in irisin-treated mice (Fig 4B). These results suggest that irisin treatment significantly alleviates the inflammatory response in the mouse model.

### Irisin inhibited the inflammatory response in ox-LDL-treated HUVECs

**Fig 5:**
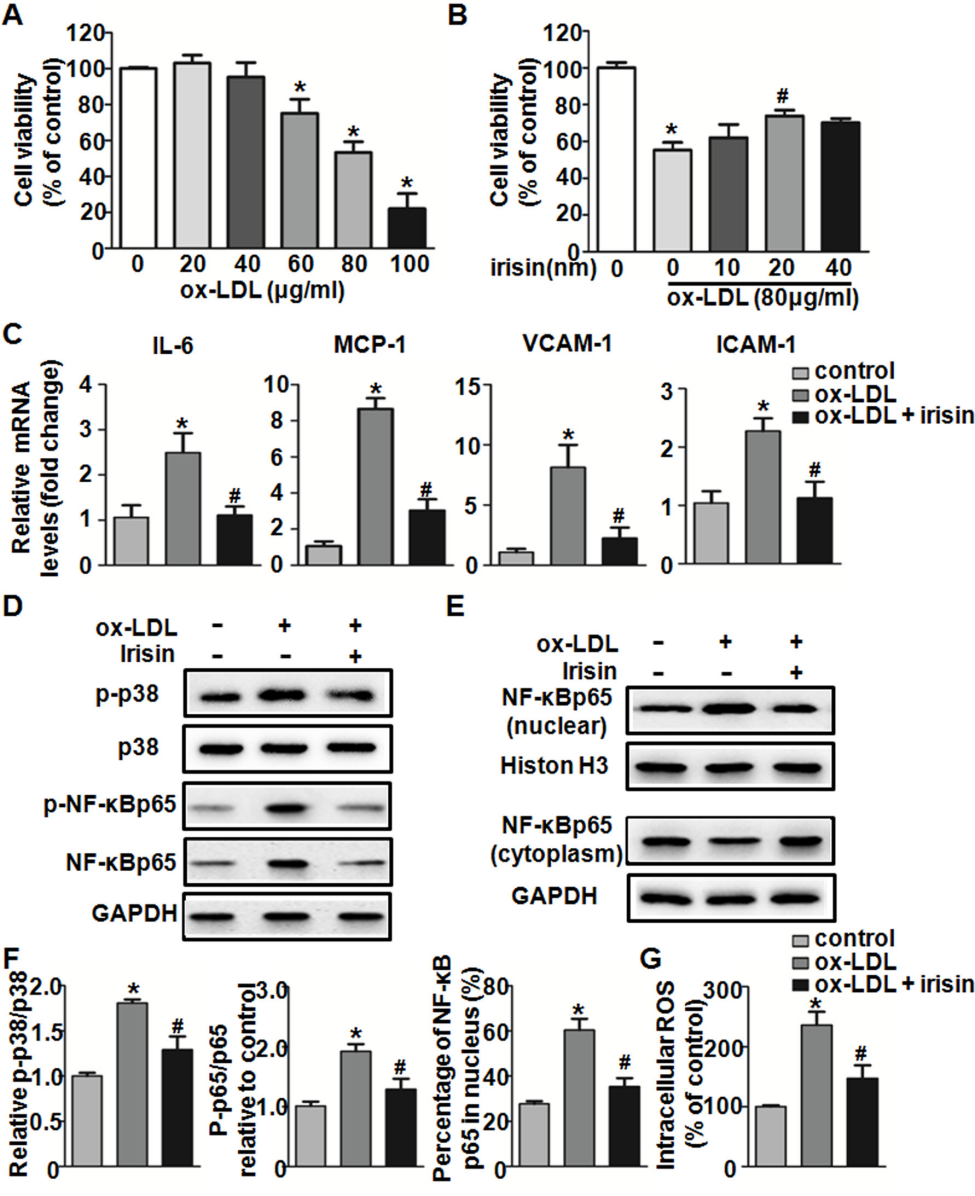
Effect of irisin on ox-LDL-induced inflammation in HUVECs. (A) HUVECs were exposed to different concentrations of ox-LDL (20–100 μg/mL) for 24h, and the cell viability was assessed through an MTT assay. (B) HUVECs were treated with different concentrations of irisin (10, 20, and 40 nM) with or without 80 μg/mL ox-LDL for 24 h, and the cell viability was assessed through an MTT assay. (C) HUVECs were incubated with irisin and ox-LDL for 24 h. The expression levels of ICAM-1, VCAM-1, MCP-1, and IL-6 were determined using qRT-PCR. The mRNA levels were normalized to that of β-actin. (D) HUVECs were cultured with irisin for 1 h, followed by stimulation with ox-LDL for 30 min. The expression levels of p-p38 MAPK, p-NF-κB p65, and NF-κB p65 were analyzed by western blot. Total p38 and GAPDH were used as a loading control respectively. (E) The expression levels of NF-κB p65 in the nucleus and cytosol were analyzed by western blot. (F) Densitometric analysis of the related bands was performed. (G) HUVECs were exposed to ox-LDL and/or irisin for 24 h. Intracellular ROS levels were measured using DCFH-DA. The measured fluorescence values were expressed as a percentage of the fluorescence in control cells. The data were expressed as the mean ± SEM of three independent experiments. *P < 0.05 *vs*. control, ^**#**^ *P <* 0.05 *vs*. ox-LDL -treated group.

To evaluate the appropriate concentration of ox-LDL, we treated cells with different concentrations of ox-LDL (20, 40, 60, 80, and 100 μg/mL) for 24 h. Ox-LDL decreased cell viability in a dose-dependent manner, and cell viability reduced to 53.32% ± 5.97% when ox-LDL was 80 μg/mL, therefore, the 80 μg/mL ox-LDL concentration was selected for subsequent experiments (Fig 5A). To assess the effect of irisin on ox-LDL-induced cell injury, cells were incubated with different concentrations of irisin (10, 20, and 40 nM) in the presence of 80 μg/mL ox-LDL for 24 h. At a dose of 20 nM, irisin significantly increased cell viability from 55.25% ± 7.33% to 78.39% ± 3.19% (Fig 5B). Therefore, 20 nM irisin was used in the subsequent experiments.

To explore the effect of irisin on the inflammatory response in ox-LDL-treated HUVECs, the mRNA levels of inflammatory genes were measured by qRT-PCR. The results showed that irisin at a concentration of 20 nM significantly suppressed ox-LDL-induced up-regulation of ICAM-1, VCAM-1, IL-6, and MCP-1 mRNA expression in HUVECs (Fig 5C). Since most of these inflammatory genes are regulated by mitogen-activated protein kinases (MAPKs) and the transcription factor NF-κB, we tested the p38 MAPK phosphorylation, NF-κB p65 phosphorylation and nuclear translocation using western blot. Irisin treatment significantly suppressed the ox-LDL-induced activation of phosphorylated p38 MAPK (Fig 5D and 5F). Meanwhile, irisin also decreased both the phosphorylated and total NF-κB p65 expression, which was activated by ox-LDL. The ratio of phosphorylated- over total- NF-κB p65 was significantly decreased after irisin treatment, compared with the ox-LDL treated group (Fig 5D and 5F). We also found that irisin treatment significantly decreased the expression of NF-κB p65 in the nucleus and increased its expression in the cytosol compared with the ox-LDL treated group. Irisin significantly suppressed ox-LDL induced nuclear translocation of NF-κB as the percentage of NF-κB p65 in the nucleus was decreased (Fig 5E and 5F).

The ROS produced by ox-LDL were shown to up-regulated p38 MAPK and then led to the activation of NF-κB, which subsequently regulated downstream pro-inflammatory events [22]. Therefore, the intracellular ROS generation was evaluated. As shown in Fig 5G, exposure of HUVECs to 80 μg/mL ox-LDL for 24 h led to the accumulation of intracellular ROS. Such accumulation in the level of intracellular ROS was significantly reduced after cells were treated with irisin.

These findings indicate that irisin may inhibit ox-LDL-induced endothelial inflammation by suppressing the ROS/p38 MAPK/NF-κB pathway.

### Irisin inhibited ox-LDL-induced apoptosis in HUVECs

**Fig 6:**
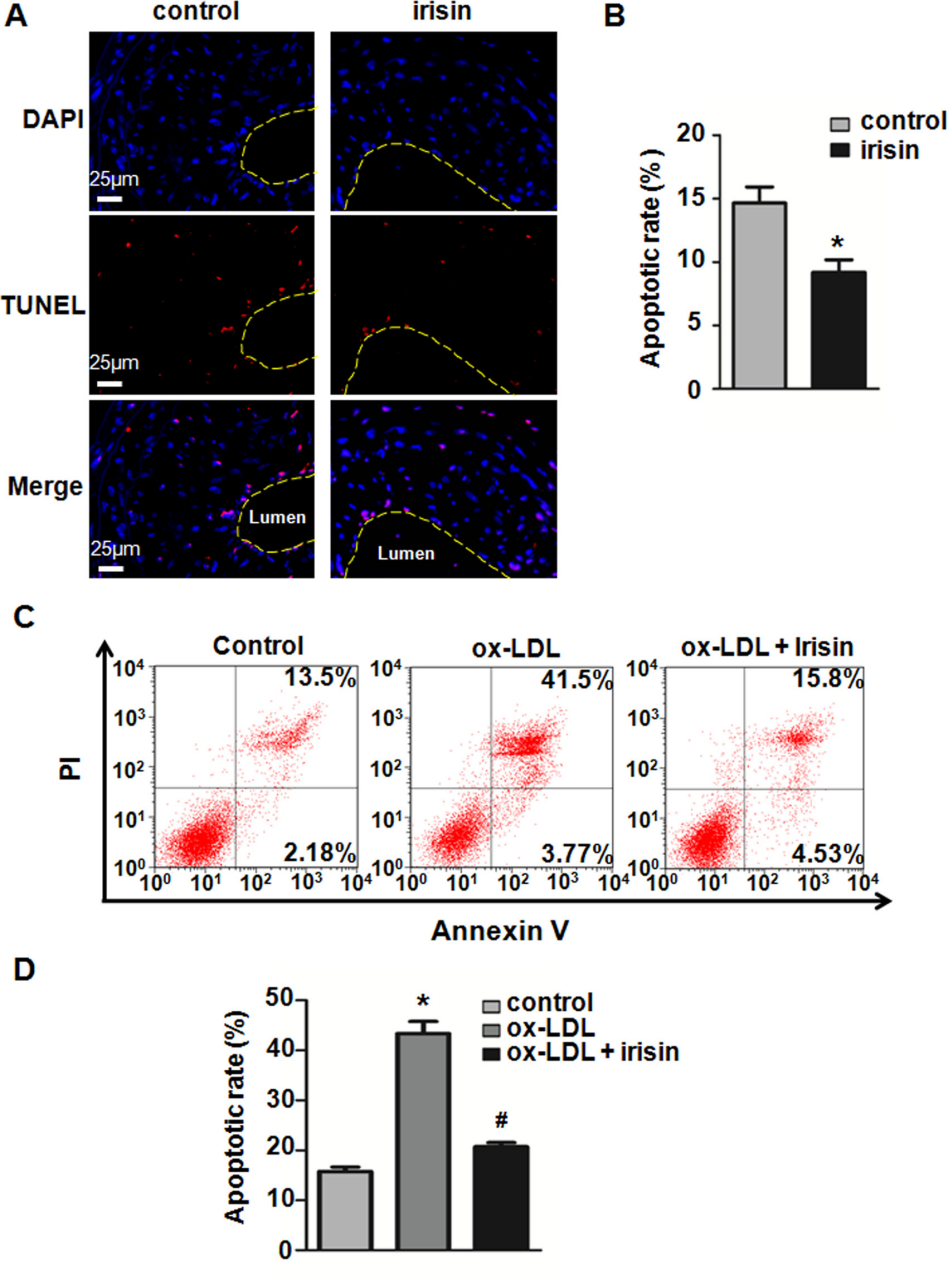
Irisin inhibited EC apoptosis in partial ligated carotid arteries and ox-LDL-induced HUVECs. (A) Sections from the mice carotid arteries were labeled by TUNEL to detect apoptotic cells and counterstained with DAPI to detect nuclei. The yellow dotted lines indicated the lumen perimeter of the vessel. (B) Quantification of TUNEL-positive cells in the plaque. (C) HUVECs were incubated with irisin and ox-LDL for 24 h. Apoptosis of ox-LDL-exposed HUVECs after treatment with irisin was detected by using flow cytometry. (D) The apoptotic rate was determined by calculating the ratio of Annexin-V-positive and Annexin-V/PI-double positive cells to total cells. The data were expressed as the mean ± SEM of three independent experiments. *P < 0.05 *vs*. control, ^**#**^ *P* < 0.05 *vs*. ox-LDL -treated group.

**Fig 7:**
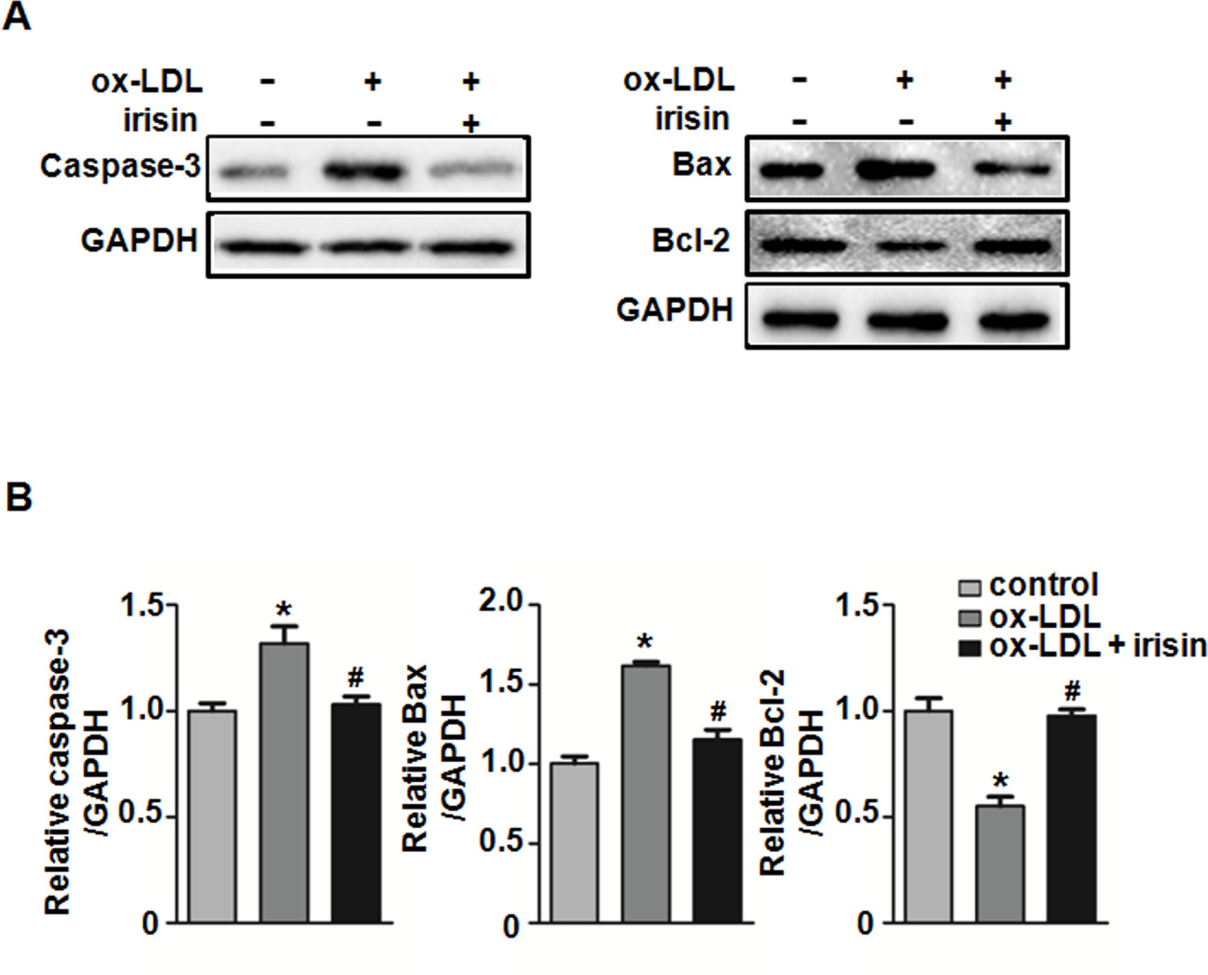
Effect of irisin on Bax, Bcl-2, and caspase-3 protein levels in ox-LDL-induced HUVECs. HUVECs were incubated with irisin and ox-LDL for 24 h. (A) The expression levels of caspase-3, Bax, and Bcl-2 were analyzed by western blot. GAPDH was used as a loading control. (B) Densitometric analysis of the related bands was performed. The data were expressed as the mean ± SEM of three independent experiments. *P < 0.05 *vs*. control, ^**#**^ *P* < 0.05 *vs*. ox-LDL -treated group.

To further understand the mechanism by which irisin administration attenuates the development of atherosclerosis, we tested whether irisin could alleviate apoptosis in atherosclerotic lesions. TUNEL staining showed that irisin treatment significantly decreased the cell apoptotic rate in atherosclerotic lesions (Fig 6A and 6B). To investigate the possible protective effect of irisin on ox-LDL-induced ECs dysfunction, Annexin V/PI staining was used to measure apoptosis. Flow cytometry results demonstrated that 80 μg/mL ox-LDL-induced cell apoptosis was effectively attenuated after being exposed to 20 nM irisin for 24 hours (Fig 6C and 6D). To further investigate the potential mechanisms, the levels of apoptosis regulator proteins caspase-3, Bcl-2, and Bax were determined using western blot. The results indicated that ox-LDL down-regulated Bcl-2 and up-regulated Bax and caspase-3 protein expression. Irisin pretreatment increased Bcl-2 and decreased Bax and caspase-3 expression and effectively repressed ox-LDL-induced pro-apoptotic events (Fig 7A and 7B).

## Discussion

The current study demonstrates that irisin can attenuate the development of aortic atherosclerotic lesions in Apo E-deficient mice. Irisin also suppress neointima formation in a carotid partial ligation mouse model. The beneficial effect of irisin in preventing plaque formation was through inhibiting inflammatory infiltration and endothelial cell apoptosis. Furthermore, we confirmed that irisin treatment inhibited the ox-LDL-induced HUVEC inflammation by suppressing the ROS/ p38 MAPK/ NF-κB signaling pathway and consequently reduced inflammatory genes expression. In addition, irisin also reduced the ox-LDL-induced HUVEC apoptosis via down-regulated Bax and caspase-3 expression and up-regulated Bcl-2 expression.

Atherosclerosis is a chronic inflammatory disease. Inflammation plays a crucial role in the etiology of atherosclerosis [4, 8]. Endothelial dysfunction is the initial step in the development of atherosclerosis [1, 23, 24]. Convincing evidence suggested that the endothelium played a vital role in the regulation of vascular inflammation [25, 26]. Irisin, a newly discovered myokine, is released by skeletal muscle after exercise and is a cleaved and secreted fragment of FNDC5. Irisin has a potent effect on the browning of white adipose tissues by stimulating expression of thermogenic genes including uncoupling protein 1 [12, 13]. Since subjects with metabolic disturbance have increased risks of cardiovascular disease and exercise improved the metabolic profile and prevented atherogenesis, we postulated that irisin played a key role in endothelial cell functional preservation [18, 27]. Very recently, Junyan Lu et al. found that systemic administration of irisin ameliorated atherosclerosis induced by diabetes in Apo E-deficient mice by suppressing high glucose-induced endothelial dysfunction and apoptosis [28]. However, the direct effect of irisin on hyperlipidemia-induced atherosclerosis and its underlying mechanisms are still unknown. In this study, we used the well-established atherosclerosis model in Apo E-deficient mice fed on a HCD to evaluate the role of irisin in endothelial function and atherogenesis. The complexity of the atherosclerotic lesions that develop in Apo E-deficient mice are similar to those described in humans and therefore represent an excellent model system for studying the pathogenesis and progression of atherosclerosis [29]. Partial carotid ligation was previously described as a model of disturbed flow that caused endothelial dysfunction and accelerated atherosclerosis. It has been extensively used in studies of vascular remodeling [30]. In our study, administration of irisin in the Apo E-deficient mice significantly attenuated the formation of atherosclerotic lesions. Similarly, the development of neointima after partial carotid ligation was also decreased after irisin treatment for 4 weeks.

Furthermore, we found that irisin decreased inflammatory cells infiltration in atherosclerotic lesions. The CD68-positive macrophage content and CD3-positive T lymphocyte content in the lesions of irisin injected mice were significantly reduced. The leukocyte recruitment required the participation of adhesion molecules and chemokines. Vascular inflammation can be triggered by many factors, and ox-LDL is one of the most significant risk factors. Once activated, vascular endothelial cells expressed adhesion molecules, which enhanced adherence of monocytes to the vascular endothelium [4]. Adhesion molecules VCAM-1 and ICAM-1 expression were increased by pro-inflammatory cytokines such as IL-1 and IL-6, which mediated the localization and recruitment of monocytes into the sub-endothelial space [31]. MCP-1, a potent monocyte attractant, bound to G-protein-coupled receptors on the surface of leukocytes targeted for activation and migration [32]. The inflammatory markers mentioned above were critical in mediating the inflammatory response and promoting atherosclerosis formation. Our study investigated the effect of irisin on the inflammatory response in Apo E-deficient mice fed on a HCD *in vivo* and cultured HUVECs exposed to ox-LDL *ex vivo*. We found that irisin inhibited the expression of MCP-1, IL-6, ICAM-1, and VCAM-1 both *in vivo* and *in vitro*. Thus, these results implied that the protective effect of irisin on atherosclerosis could be through its anti-inflammatory effects.

The expression of chemokines and adhesion molecules were regulated by a variety of intracellular signaling pathways, including MAPKs and nuclear transcriptional factors [33]. It is known that ox-LDL can activate the NF-κB, which mediated the expression of a number of pro-inflammatory molecules including IL-6, ICAM-1, and VCAM-1. Usually, NF-κB exists in the cytoplasm as a heterodimer of the p65 and p50 subunits. Stimulation by pro-inflammatory cytokines leads to the liberation of p65 and p50, which can then translocate to the nucleus to affect target gene expression [34]. In the present study, we found that irisin can significantly attenuate the nuclear translocation of NF-κB p65 which was activated by ox-LDL in HUVECs. Furthermore, accumulating evidence has demonstrated that the phosphorylation of the p65 subunit is critical for binding to its target sites on DNA [35]. It was important to verify whether irisin played a role in the phosphorylation of NF-κB p65. The results showed that irisin decreased the p-p65 level. This may be an important mechanism in which irisin could regulate the production of MCP-1, IL-6, ICAM-1, and VCAM-1. In addition, we confirmed that ox-LDL-induced phosphorylation of the p38 MAPK pathway was suppressed by irisin treatment. This pathway has been identified as a major element upstream of the NF-κB pathway and was essential to the ox-LDL-induced and NF-κB-dependent gene expression [33]. Accumulating evidence indicated that ox-LDL-induced ROS formation played an important role in the mediation of endothelial dysfunction and thus regulated endothelial inflammation [36]. The ox-LDL-induced ROS was shown to up-regulate p38 MAPK and then led to the activation of NF-κB, which subsequently regulated downstream pro-inflammatory events. For these reason, we postulated that ROS might be participating in the inhibitory effect of irisin on ECs inflammation. Consistent with our hypothesis, ox-LDL-induced intracellular ROS generation was significantly reduced after treated the HUVECs with irisin. We can conclude, at least in part, that irisin improved ox-LDL-induced endothelial inflammation through the ROS/ p38 MAPK/ NF-κB signaling pathway.

Ox-LDL induced apoptosis in vascular endothelial cells is the pathogenic basis of atherosclerosis. In the present study, we demonstrated the protective effect of irisin on ox-LDL induced apoptosis in HUVECs. The Bcl-2 family played an essential role in regulating the apoptosis involved in the endogenous apoptosis pathway. The pro-apoptotic protein Bax and the anti-apoptotic protein Bcl-2 are both members of the Bcl-2 family [37]. Caspase-3 also plays a key role in apoptosis execution [38]. We found that irisin suppressed Bax and promoted the Bcl-2 expression in ox-LDL treated HUVECs. Irisin also reduced the caspase-3 expression in ox-LDL-induced HUVECs.

In addition, macrophage-derived foam cell formation by uptake ox-LDL was a key determinant of atherosclerotic lesion formation. We also determined the effects of irisin on ox-LDL-induced foam cell formation [39]. The Oil-Red O staining showed that incubation of RAW 264.7 cells with 80 μg/mL ox-LDL for 24 hours resulted in foam cell formation. Interestingly, ox-LDL-induced foam cell formation was inhibited by irisin treatment (S2 Fig). Further studies are needed to clarify the underlying mechanisms involved in the inhibitory effects of irisin on foam cell formation.

Accumulating evidence had demonstrated that moderate-intensity physical activity suppresses cardiovascular morbidity and mortality in the whole population [40–42]. Physical activity or routine exercise is inversely related to the extent of atherosclerosis and the primary and secondary incidence of cardiovascular events [43, 44]. However, the mechanisms underlying the benefits of exercise in the prevention of atherosclerosis largely remain to be established. A recent study using tandem mass spectrometry demonstrated that human irisin existed, circulated, and was regulated by exercise [14]. As irisin is a myokine released by skeletal muscle after exercise, our results may provide the mechanistic link between exercise and exercise-based health benefits in cardiovascular disease prevention.

In conclusion, our study demonstrated that irisin can attenuate the development of atherosclerosis and neointima formation in the Apo E-deficient mouse model. This finding indicates that irisin can serve as a potential therapeutic target in the treatment of atherosclerosis.

## Supporting Information

S1 Fig The purity of r-irisin used in this study. The secreted r-irisin was separated by SDS-PAGE and stained with coomassie brilliant blue.
(TIF)

S2 Fig Effect of irisin on ox-LDL induced foam cell formation in RAW 264.7 cells. (A) RAW 264.7 cells were exposed to 80 μg/mL ox-LDL in the presence or absence of 20 nM irisin for 24 h. Representative photographs showing RAW 264.7 cells stained with Oil-Red O. (B) Oil-Red-positive RAW 264.7 cells were quantified. The data were expressed as the mean ± SEM of three independent experiments. *P < 0.05 *vs*. control, ^**#**^ *P* < 0.05 *vs*. ox-LDL -treated group.
(TIF)

S1 Table Primer pairs used for gene expression analysis. (DOCX)

S2 Table Body weight and lipid profiles of mice at the end of study. (DOCX)
