# Supplementary figures and images for: Protective Effect of Irisin on Atherosclerosis via Suppressing Oxidized Low Density Lipoprotein Induced Vascular Inflammation and Endothelial Dysfunction
Source: PLoS One. 2016 Jun 29;11(6):e0158038. doi: 10.1371/journal.pone.0158038 (PMC4927070; doi:10.1371/journal.pone.0158038)

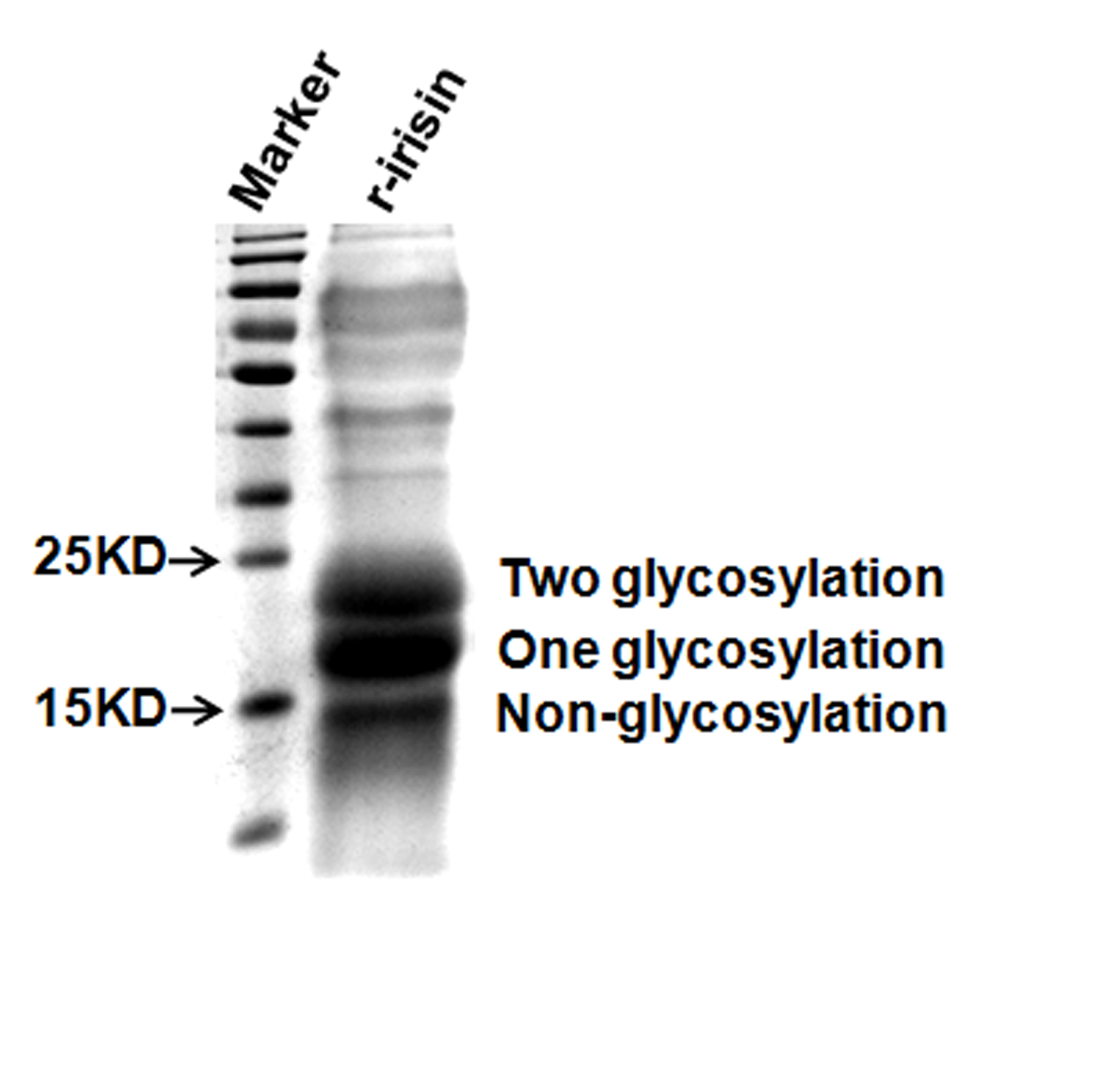

Supplement: S1 Fig — The secreted r-irisin was separated by SDS-PAGE and stained with coomassie brilliant blue. (TIF) [file pone.0158038.s001.tif]

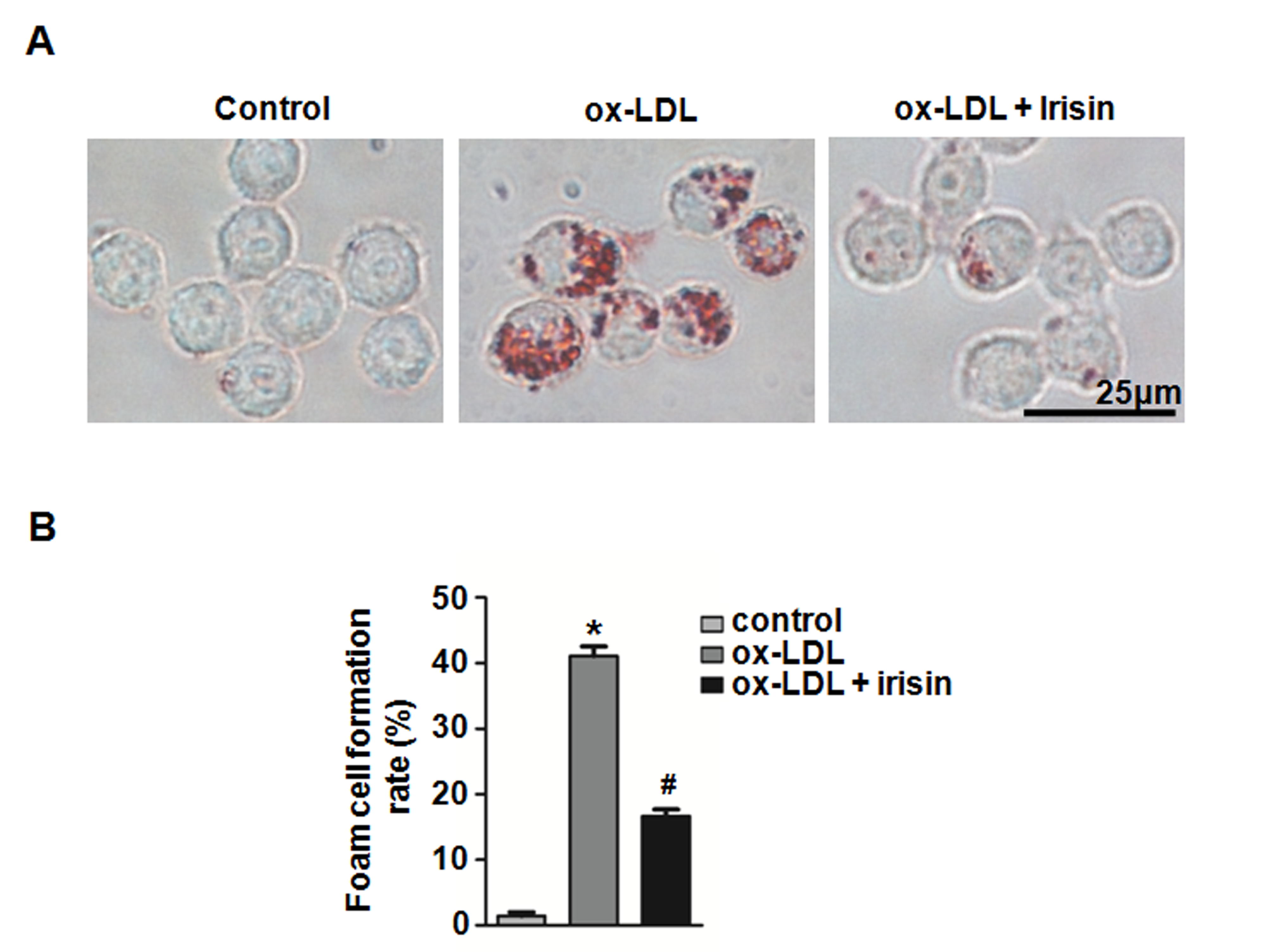

Supplement: S2 Fig — (A) RAW 264.7 cells were exposed to 80 μg/mL ox-LDL in the presence or absence of 20 nM irisin for 24 h. Representative photographs showing RAW 264.7 cells stained with Oil-Red O. (B) Oil-Red-positive RAW 264.7 cells were quantified. The data were expressed as the mean ± SEM of three independent experiments. *P < 0.05 vs. control, # P < 0.05 vs. ox-LDL -treated group. (TIF) [file pone.0158038.s002.tif]
