# Supplementary material for: Protective Effect of Irisin on Atherosclerosis via Suppressing Oxidized Low Density Lipoprotein Induced Vascular Inflammation and Endothelial Dysfunction
Source: PLoS One. 2016 Jun 29;11(6):e0158038. doi: 10.1371/journal.pone.0158038 (PMC4927070; doi:10.1371/journal.pone.0158038)
Supplement: S1 Table — (DOCX) [file pone.0158038.s003.docx]

**S1 Table**

| Genes | Forward primer(5’-3’) | Reverse primer(5’-3’) |
| --- | --- | --- |
| human β-actin | ATCATGTTTGAGACCTTCAACA | CATCTCTTGCTCGAAGTCCA |
| human IL-6  human MCP-1  human ICAM-1  human VCAM-1 | TTCTCCACAAGCGCCTTCGGTCCA  GCTCATAGCAGCCACCTTCATTC  CCCATGAAACCGAACACAC  TCTCATTGACTTGCAGCACC | AGGGCTGAGATGCCGTCGAGGATGTA  GGACACTTGCTGCTGGTGATTC  ACTCTGTTCAGTGTGGCACC  CTCATTCGTCACCTTCCCAT |
| mouse β-actin  mouse IL-6  mouse MCP-1  mouse ICAM-1  mouse VCAM-1 | GAAATCGTGCGTGACATCAAAG  AGCCAGAGTCCTTCAGAGAGATAC  CTCACCTGCTGCTACTCATTCAC  GTGATGCTCAGGTATCCATCCA  GTTCCAGCGAGGGTCTACC | TGTAGTTTCATGGATGCCACAG  AATTGGATGGTCTTGGTCCTTAGC  ATGTCTGGACCCATTCCTTCTTG  CACAGTTCTCAAAGCACAGCG  AACTCTTGGCAAACATTAGGTGT |
