# Supplementary material for: Protective Effect of Irisin on Atherosclerosis via Suppressing Oxidized Low Density Lipoprotein Induced Vascular Inflammation and Endothelial Dysfunction
Source: PLoS One. 2016 Jun 29;11(6):e0158038. doi: 10.1371/journal.pone.0158038 (PMC4927070; doi:10.1371/journal.pone.0158038)
Supplement: S2 Table — (DOCX) [file pone.0158038.s004.docx]

**S2 Table**

| Parameters | control | irisin |
| --- | --- | --- |
| Body weight (g)  TC (mmol/L)  TG(mmol/L)  LDL(mmol/L)  HDL(mmol/L) | 29.01±0.64  22.68±1.28  2.84±0.79  4.74±1.22  2.74±0.88 | 28.03±0.92  21.95±2.54  2.73±0.63  5.68±1.53  2.35±0.90 |

Data are represented as mean ± SEM.

control: HCD-fed Apo E-deficient mice injected with NS, irisin: HCD-fed Apo E-deficient mice injected with 0.5μg/g body weight/day irisin.
